# Supplementary material for: Rapid molecular detection of respiratory pathogens in patients admitted with suspected community-acquired pneumonia: secondary analysis of a randomized controlled trial
Source: Microbiol Spectr. 2025 Aug 12;13(9):e01260-25. doi: 10.1128/spectrum.01260-25 (PMC12403596; doi:10.1128/spectrum.01260-25)
Supplement: Tables S1 to S4 — Table S1: Interpretation Guideline. Table S2: Targets of the Biofire FilmArray Pneumonia Panel plus. Table S3: Classification of "targeted, adequate and inappropriate" treatment. Table S4: Classification of antibiotic treatment based on CAP pathogens. [file spectrum.01260-25-s0001.docx]

**Supplemental material**

**Title:** Rapid molecular detection of respiratory pathogens in patients admitted with suspected community-acquired pneumonia: Secondary analysis of a randomized controlled trial

**Authors:** Mariana B. Cartuliares ^1, 2*^, Helene Skjøt-Arkil ^1,2^, Christian B. Mogensen ^1, 2^, Steen L. Andersen ^3^, and Flemming S. Rosenvinge ^4,5^

**Affiliations:**

^1^ University Hospital of Southern Denmark, Department of Emergency Medicine, 6200 Aabenraa, Denmark;

^2^ University of Southern Denmark, Department of Regional Health Research, 6200 Aabenraa, Denmark

^3^ University Hospital of Southern Denmark, Department of Clinical Microbiology, 6200 Aabenraa, Denmark

^4^ Odense University Hospital, Department of Clinical Microbiology, 5000 Odense C, Denmark

^5^ University of Southern Denmark, Research Unit of Clinical Microbiology, 5000 Odense C, Denmark*Corresponding author email: mbc@rsyd.dk

**Table of contents**

S1: Interpretation Guideline …………………….…………………………………………………………………………….2

Table S2: Targets of the Biofire® FilmArray® Pneumonia Panel plus…………………………………….….6

Table S3: Classification of ”targeted, adequate and inappropriate” treatment…………………..……7

Table S4: Classification of antibiotic treatment based on CAP pathogens………………………………..8

S1: Interpretation Guideline

**Guidance of results from POC-PCR**

***FilmArray® Pneumonia Panel plus***


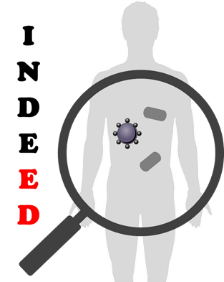


This guidance is developed to the INDEED-study (Infectious diseases in Emergency Department).

Emergency department physicians from Hospital Sønderjylland in Aabenraa, Hospital Lillebælt in Kolding, and Odense University Hospital in Odense, will receive this action card along with the results from sputum sample analyses.

In case of doubt in the interpretation of the results, the physician is encouraged to contact the local clinical microbiologist.

| **Agens** | **Association with CAP^#^** | **Remarks** | **Antibiotics** | |
| --- | --- | --- | --- | --- |
|  |  |  | **First choice** | **Penicillin allergy** |
| *Streptococcus pneumoniae** | Frequent and likely pathogen | Part of the normal microbiota in upper respiratory tract.  May be contamination with pharyngeal microbiota. | Benzylpenicillin 1.2g (2 mill.IE) x4 i.v.  *or*  Phenoxymethylpenicillin 0.6g (1 mill.IE) x4 oral | Cefuroxime 1.5g x 3 i.v.  *or*  Roxithromycin 300mg x1 oral |
| *H. Influenzae influenza** | Frequent and likely pathogen |  | Ampicillin 2g x4 i.v.  *or*  Benzylpenicillin 1.2g (2 mill. IE) x4 i.v.  *or*  Piv-ampicillin 1g x3 oral  *or*  Amoxicillin 1g x3 oral | Cefuroxime 1.5g x 3 i.v.  *or*  Doxycycline 100mg x2 first 24 hours oral followed by 100mg x1 oral |
| *Streptococcus pyogenes** | Probable, but rare pathogen | Part of the normal microbiota in upper respiratory tract.  These pathogens relatively often represent contamination with pharyngeal microbiota.  Infection caused by *Streptococcus pyogenes* or *Staphylococcus aureus* will usually results in severe pneumonia. | Benzylpenicillin  1.2g (2 mill. IE) x4 i.v. | Cefuroxime 1.5g x3 i.v. |
| *Streptococcus agalactiae** | Rare pathogen in adults |  | Benzylpenicillin 1.2g (2 mill. IE) x4 i.v. | Cefuroxime 1.5g x3 i.v. |
| *Staphylococcus aureus** | Probable, but rare pathogen |  | Cloxacillin 1g x4 i.v. | Cefuroxime 1.5g x3 i.v. |
| *Moraxella catarrhalis** | Probable pathogen |  | Piperacillin-tazobactam  4/0.5g x3 i.v.  *or*  amoxicillin-clavulanic acid 500/125mg x3 oral | Cefuroxime 1.5g x3 i.v.  *or*  Roxithromycin 300mg x1 oral  *or*  Azithromycin 500mg x1 oral |
|  | | | | |
| *Legionella pneumophila*  *Mycoplasma pneumonia* | Likely causative pathogen | Is not a part of the normal respiratory microbiota. | Azithromycin 500mg x1 i.v./oral | |
| *Chlamydia pneumoniae* | Probable causative pathogen | Is not a part of the normal respiratory microbiota  Will usually cause mild infections. In case of severe infection, other pathogens/super-infection should be considered. | Azithromycin 500mg x1 i.v./oral | |

| **Agens** | **Association with CAP^#^** | **Remarks** | **Antibiotics** |
| --- | --- | --- | --- |
| *Pseudomonas aeruginosa**  *Acinetobacter calcoaceticus-baumannii complex**  *Enterobacter cloacae**  *Escherichia coli**  *Klebsiella (Enterobacter) aerogenes**  *Klebsiella oxytoca**  *Klebsiella pneumoniae group**  *Proteus**  *Serratia marcescens** | Very rare causative pathogens | These findings usually represents colonization. | These findings should typically not lead to adjustment of empirical antimicrobial treatment. |
|  | | | |
| Influenza A  Influenza B | Frequent pathogens | Is not a part of the normal respiratory microbiota Bacterial superinfection can occur. | Consider whether the patient's pneumonia symptoms can be explained by viral infection, and whether antibiotic treatment is necessary / indicated. |
| Parainfluenza virus  Respiratory Syncytial  Adenovirus  Coronavirus  *(does not include SARS-CoV-2)*  Human Rhinovirus/Enterovirus  Human Metapneumovirus | Probable pathogens | Usually causes mild infections. In case of severe infection, other pathogens / superinfection should be considered.  May be an accidental finding due to previous /recent / asymptomatic infection. |  |
|  | | | |
| Not detected  *(POC-PCR(FilmArray) is negative)* | A negative result does not rule out pneumonia, but means that CAP caused by the most common pathogens is less likely. Consider whether the pneumonia diagnosis is correct and consider investigation for rare causes of pneumonia (e.g. tubeculosis or *Chlamydia psittaci*). | | |
|  | | | |

#CAP: Community-Acquired Pneumonia

*: Concentration (copies/mL) is reported in the POC-PCR (FilmArray) result

Most bacterial causative pathogens of CAP are also part of the normal respiratory microbiota or may colonize the upper respiratory tract, and the clinical relevance of these findings must always be assessed carefully.

For the bacterial agents marked with “*”, a concentration (copies/mL) is reported in the POC-PCR (FilmArray) result. There is a reasonable correlation between copies/mL and the culture-based measure “CFU/mL”, however, “copies/mL” is typically a factor of 10-100 higher than the corresponding “CFU/mL”.

The limits of significance are not well established and depend probably on the agent, the quality of the sample and the clinical context - and must therefore be used with caution. The Infectious Diseases Society of America and the American Society of Microbiology^1^ propose the following culture-based limits for hospital-acquired pneumonia:

| Culture-based measure | POC-PCR (FilmArray) concentration | Interpretation (caution) |
| --- | --- | --- |
| < 10^4^ CFU/mL | ≈ < 10^5^ copies/mL | Indicates mixture with normal flora |
| 10^4^ – 10^5^ CFU/mL | ≈ 10^5^-10^6^ copies/mL | Gray zone |
| > 10^5^ CFU/mL | ≈ >10^6^ copies/mL | Indicates real findings |

Developed by microbiologist Flemming Rosenvinge, Department of Clinical Microbiology, Odense University Hospital in Odense, and microbiologist Claus Østergaard, Department of Clinical

Microbiology, Hospital Lillebælt in Kolding, Denmark

*Version 1.1 – February 7th 2021*

*^1^ Miller, J. M., Binnicker, M. J., Campbell, S., et al. A Guide to Utilization of the Microbiology Laboratory for Diagnosis of Infectious Diseases: 2018 Update by the Infectious Diseases Society of America and the American Society for Microbiology. Clinical Infectious Diseases, 67(6), e1–e94. https://doi.org/10.1093/cid/ciy381*

Table S2: Targets of the Biofire® FilmArray® Pneumonia Panel plus (Biomérieux, Marcy l’Etoile, France)

| **Bacteria** | **Atypical bacteria** | **Antimicrobial resistance genes** |
| --- | --- | --- |
| *Acinetobacter calcoaceticus baumannii complex* | *Chlamydophilia pneumoniae* | *mecA/C and MREJ* |
| *Enterobacter cloacae complex* | *Legionella pneumophila* | *KPC,* |
| *Escherichia coli* | *Mycoplasma pneumonia* | *CTX-M* |
| *H. Influenzae influenzae* |  | *NDM* |
| *Klebsiella aerogenes* |  | *Oxa48-like* |
| *Klebsiella oxytoca* | **Viruses** | *VIM* |
| *Klebsiella pneumoniae group* | *Influenza A* | *IMP* |
| *Moraxella catarrhalis* | *Influenza B* |  |
| *Proteus* | *Adenovirus** |  |
| *Pseudomonas aeruginosa* | *Parainfluenza virus* |  |
| *Serratia marcescens* | *Coronavirus (CoV)*** |  |
| *Staphylococcus aureus* | *Human metapneumovirus* |  |
| *Streptococcus agalactiae,* | *Human rhinovirus/enterovirus* |  |
| *Streptococcus pneumoniae* | *MERS-CoV* |  |
| *Streptococcus pyogenes* | *Respiratory syncytial virus* |  |

* Adenovirus is not included in our analysis due to the expiration date specific for Adenovirus announced
by Biofire Nordic 21. July 2021. Biofire® FilmArray® Pneumonia plus (PN plus) Panel (RFIT-ASY-0142 and RFIT-ASY-0143).
** Coronavirus (CoV): variants (229E, OC43, HKU1, NL63)

| Table S3: Classification of ”targeted, adequate and inappropriate” treatment | | | | | | | |
| --- | --- | --- | --- | --- | --- | --- | --- |
| **Antimicrobial** | **Microbiological agents** | | | | | | |
|  | *S. pneumoniae* | *H. influenzae* | *M. catarrhalis* | *P. aeruginosa* | *S. aureus* | Hemolytic streptococci | *L. pneumophila* |
| Benzylpenicillin | Targeted | Targeted | Inappropriate | Inappropriate | Inappropriate | Targeted | Inappropriate |
| Phenoxymethyl-  penicillin | Targeted | Inappropriate | Inappropriate | Inappropriate | Inappropriate | Targeted | Inappropriate |
| Ampicillin | Adequate | Targeted | Inappropriate | Inappropriate | Inappropriate | Adequate | Inappropriate |
| Pivampicillin | Adequate | Targeted | Inappropriate | Inappropriate | Inappropriate | Adequate | Inappropriate |
| Amoxicillin | Adequate | Targeted | Inappropriate | Inappropriate | Inappropriate | Adequate | Inappropriate |
| Pivmecillinam | Inappropriate | Inappropriate | Inappropriate | Inappropriate | Inappropriate | Inappropriate | Inappropriate |
| Mecillinam | Inappropriate | Inappropriate | Inappropriate | Inappropriate | Inappropriate | Inappropriate | Inappropriate |
| Dicloxacillin | Inappropriate | Inappropriate | Inappropriate | Inappropriate | Targeted | Inappropriate | Inappropriate |
| Cloxacillin | Inappropriate | Inappropriate | Inappropriate | Inappropriate | Targeted | Inappropriate | Inappropriate |
| Flucloxacillin | Inappropriate | Inappropriate | Inappropriate | Inappropriate | Targeted | Inappropriate | Inappropriate |
| Amoxicillin/  Clavulansyre | Adequate | Targeted | Targeted | Inappropriate | Adequate | Adequate | Inappropriate |
| Tazobactam/  Piperacillin | Adequate | Targeted | Targeted | Targeted | Adequate | Adequate | Inappropriate |
| Cefuroxime | CAVE/Targeted | CAVE/Targeted | Targeted | Inappropriate | CAVE/Targeted | CAVE/Targeted | Inappropriate |
| Cefotaxim | Adequate | Adequate | Adequate | Inappropriate | Adequate | Adequate | Inappropriate |
| Ceftriaxon | Adequate | Adequate | Adequate | Inappropriate | Adequate | Adequate | Inappropriate |
| Ceftazidim | Inappropriate | Adequate | Adequate | Targeted | Inappropriate | Adequate | Inappropriate |
| Cefepime | Adequate | Adequate | Adequate | Adequate | Adequate | Adequate | Inappropriate |
| Meropenem | Adequate | Adequate | Adequate | Adequate | Adequate | Adequate | Inappropriate |
| Ertapenem | Adequate | Adequate | Adequate | Inappropriate | Adequate | Adequate | Inappropriate |
| Imipenem and cilastatin | Adequate | Adequate | Adequate | Adequate | Adequate | Adequate | Inappropriate |
| Macrolides* | CAVE/Targeted | Inappropriate | Targeted | Inappropriate | Inappropriate | CAVE/Targeted | Targeted |
| Clindamycin | CAVE/Targeted | Inappropriate | Inappropriate | Inappropriate | CAVE/Targeted | CAVE/Targeted | Inappropriate |
| Doxycylin | Adequate | CAVE/Targeted | Adequate | Inappropriate | Adequate | Adequate | Targeted |
| Tetracyclin | Adequate | CAVE/Targeted | Adequate | Inappropriate | Adequate | Adequate | Targeted |
| Tigecyclin | Adequate | Adequate | Adequate | Inappropriate | Adequate | Adequate | Inappropriate |
| Tobramycin | Inappropriate | Inappropriate | Inappropriate | Targeted | Inappropriate | Inappropriate | Inappropriate |
| Gentamicin | Inappropriate | Inappropriate | Inappropriate | Targeted | Inappropriate | Inappropriate | Inappropriate |
| Ciprofloxacin | Inappropriate | CAVE/Targeted | Adequate | Targeted | Inappropriate | Inappropriate | Targeted |
| Moxifloxacin | Adequate | Adequate | Adequate | Inappropriate | Adequate | Adequate | Targeted |
| Trimethoprim | Inappropriate | Inappropriate | Inappropriate | Inappropriate | Inappropriate | Inappropriate | Inappropriate |
| Sulfamethizol | Inappropriate | Inappropriate | Inappropriate | Inappropriate | Inappropriate | Inappropriate | Inappropriate |
| Sulfamethoxazole  and trimethoprim | Adequate | Adequate | Adequate | Inappropriate | Adequate | Adequate | Inappropriate |

Green (targeted treatment): Antibiotics directed against a bacterial pathogen detected by culture without being unnecessary board-spectrum.
Blue (CAVE/Targeted): Considered targeted treatment if the patient was registered as allergic to penicillins.
Yellow (Adequate): Antibiotics that are active against the bacterial pathogen detected by culture.
Orange (Inappropriate): Antibiotics that are not recommended and/or regarded inactive against the bacterial pathogen detected by culture.
*Macrolides: Erythromycin or roxithromycin or clarithromycin or azithromycin.

| Table S4: Classification of antibiotic treatment of targeted, adequate and inappropriate treatment based CAP pathogens | | | |
| --- | --- | --- | --- |
| **Microorganisms (109 detected by POC-PCR)** | **Treatment** | **Classification based on  pneumococci, H. influenzae,  M. catarrhalis, P. aeruginosa  and S. aureus** | **Classification based on pneumococci and H. influenzae** |
| **Considered one pathogen** |  |  |  |
| *S. pneumoniae* | Benzylpenicillin | Targeted | Targeted |
| *S. pneumoniae* | Benzylpenicillin | Targeted | Targeted |
| *H. Influenzae* | Benzylpenicillin | Targeted | Targeted |
| *H. Influenzae* | Benzylpenicillin | Targeted | Targeted |
| *H. Influenzae, S. agalactiae* | piperacillin-tazobactam | Targeted | Targeted |
| *H. Influenzae* | pivmecillinam/pivampicillin/ampicillin | Targeted | Targeted |
| *H. Influenzae* | Benzylpenicillin | Targeted | Targeted |
| *H. Influenzae*, Human metapneumovirus | piperacillin-tazobactam | Targeted | Targeted |
| *H. Influenzae*, Human metapneumovirus | Benzylpenicillin | Targeted | Targeted |
| *H. Influenzae*, Rhinovirus/enterovirus | ampicillin/mecillinam | Targeted | Targeted |
| *H. Influenzae* | benzylpenicillin | Targeted | Targeted |
| *H. Influenzae* | pivmecillinam/pivampicillin/ampicillin | Targeted | Targeted |
| *H. Influenzae*, Rhinovirus/enterovirus | pivmecillinam/pivampicillin/ampicillin | Targeted | Targeted |
| *H. Influenzae* | Benzylpenicillin | Targeted | Targeted |
| *H. Influenzae,* Rhinovirus/enterovirus, *E.coli* | pivmecillinam/pivampicillin/ampicillin | Targeted | Targeted |
| *H. Influenzae*, Rhinovirus/enterovirus | Benzylpenicillin | Targeted | Targeted |
| *H. Influenzae* | pivmecillinam/pivampicillin/ampicillin | Targeted | Targeted |
| *H. Influenzae*, Rhinovirus/enterovirus | pivmecillinam/pivampicillin/ampicillin | Targeted | Targeted |
| *H. Influenzae*, Human metapneumovirus | CAVE, tetracyclins, cephosporins | Targeted | Targeted |
| *H. Influenzae, S.agalactiae,* Respiratory syncytial virus | Benzylpenicillin | Targeted | Targeted |
| *H. Influenzae* | Benzylpenicillin | Targeted | Targeted |
| *H. Influenzae* | piperacillin-tazobactam | Targeted | Targeted |
| *H. Influenzae* | Benzylpenicillin | Targeted | Targeted |
| *H. Influenzae* | CAVE, cephalosporiner | Targeted | Targeted |
| *H. Influenzae*, Rhinovirus/enterovirus | Benzylpenicillin | Targeted | Targeted |
| *H. Influenzae*, Corona virus | Benzylpenicillin | Targeted | Targeted |
| *H. Influenzae* | benzylpenicillin, macrolides | Adequate | Adequate |
| *H. Influenzae,* Rhinovirus/enterovirus | Cephalosporins | Adequate | Adequate |
| *H. Influenzae* | benzylpenicillin, macrolides | Adequate | Adequate |
| *H. Influenzae* | benzylpenicillin, macrolides | Adequate | Adequate |
| *H. Influenzae* | benzylpenicillin, macrolides | Adequate | Adequate |
| *S. pneumoniae, E. coli* | ampicillin/mecillinam | Adequate | Adequate |
| *S. pneumoniae*, Corona virus | piperacillin-tazobactam | Adequate | Adequate |
| *H. Influenzae* | phenoxymethylpenicillin | Inappropriate | Inappropriate |
| *H. Influenzae* | phenoxymethylpenicillin | Inappropriate | Inappropriate |
| *H. Influenzae* | CAVE, macrolides | Inappropriate | Inappropriate |
| *H. Influenzae* | Ingen beh | Inappropriate | Inappropriate |
| *H. Influenzae* | Ingen beh | Inappropriate | Inappropriate |
| *H. Influenzae,* Corona virus | Ingen beh | Inappropriate | Inappropriate |
| *H. Influenzae* | CAVE, ingen beh | Inappropriate | Inappropriate |
| *M. catarrhalis,* Respiratory syncytial virus | phenoxymethylpenicillin | Inappropriate | # |
| *M. catarrhalis* | ingen beh | Inappropriate | # |
| *M. catarrhalis* | CAVE, ingen beh | Inappropriate | # |
| *M. catarrhalis* | amoxicillin-clavulanic acid | Targeted | # |
| *M. catarrhalis* | piperacillin-tazobactam | Targeted | # |
| *S. aureus* | CAVE and macrolides | Inappropriate | # |
| *S. aureus* | CAVE, benzylpenicillin | Inappropriate | # |
| *S. aureus* | pivmecillinam/pivmecillinam/ampicillin | Inappropriate | # |
| *S. aureus* | benzylpenicillin | Inappropriate | # |
| *S. aureus* | benzylpenicillin | Inappropriate | # |
| *S. aureus* | phenoxymethylpenicillin | Inappropriate | # |
| *S. aureus* | ingen beh | Inappropriate | # |
| *S. aureus* | ingen beh | Inappropriate | # |
| *S. aureus*, Respiratory syncytial virus | ingen beh | Inappropriate | # |
| *S. aureus* | ingen beh | Inappropriate | # |
| *S. aureus* | ingen beh | Inappropriate | # |
| *S. aureus*, Parainfluenza virus | amoxicillin-clavulanic acid | Adequate | # |
| *S. aureus, S. marcescens, E. coli* | piperacillin-tazobactam | Adequate | # |
| *S. aureus, S. marcecens* | amoxicillin-clavulanic acid | Adequate | # |
| **Considered ≥ two pathogens** | | | |
| *H. Influenzae, S. pneumoniae* | benzylpenicillin | Targeted | Targeted |
| *H. Influenzae, s. pneumoniae* | benzylpenicillin | Targeted | Targeted |
| *H. Influenzae, s. pneumoniae* | benzylpenicillin | Targeted | Targeted |
| *H. Influenzae, S. agalactiae, S. pyrogenes*  *S. pneumoniae* | benzylpenicillin | Targeted | Targeted |
| *H. Influenzae, S. pneumoniae* | benzylpenicillin | Targeted | Targeted |
| *H. Influenzae, S. pneumoniae,* Rhinovirus/enterovirus | benzylpenicillin | Targeted | Targeted |
| H. Influenzae, S. pneumoniae, Rhinovirus/enterovirus | CAVE, cephalosporins | Targeted | Targeted |
| *H. Influenzae, M. catarrhalis, S. pneumoniae* | phenoxymethylpenicillin | Inappropriate | Inappropriate |
| *H. Influenzae, S. aureus, S. pneumoniae* | piperacillin-tazobactam | Adequate | Targeted |
| *M. catarrhalis, S. pneumoniae* | piperacillin-tazobactam | Targeted | Adequate |
| *M. catarrhalis, S. aureus, S. pneumoniae* | piperacillin-tazobactam | Adequate | Adequate |
| *H. Influenzae and S. agalactiae, S. penumoniae* | benzylpenicillin, macrolides | Adequate | Adequate |
| *H. Influenzae, S. aureus*, Rhinovirus/enterovirus | pivmecillinam/pivampicillin/ampicillin | Inappropriate | Targeted |
| *H. Influenzae, S. aureus*, Rhinovirus/enterovirus | pivmecillinam/pivampicillin/ampicillin | Inappropriate | Targeted |
| *H. Influenzae, S. aureus* | benzylpenicillin | Inappropriate | Targeted |
| *H. Influenzae, S. aureus* | benzylpenicillin | Inappropriate | Targeted |
| *H. Influenzae, K. oxytoca, P. aeruginosa,*  *S. aureus* | CAVE, amoxicillin-clavulanic acid | Inappropriate | Targeted |
| *H. Influenzae, M. catarrhalis* | CAVE, ingen beh | Inappropriate | Inappropriate |
| *H. Influenzae and S. aureus* | aminoglycosider | Inappropriate | Inappropriate |
| *H. Influenzae, M. catarrhalis,* Corona virus | piperacillin-tazobactam | Targeted | Targeted |
| *H. Influenzae, E. coli, M. catarrhalis, Proteus, S. aureus* | piperacillin-tazobactam | Adequate | Targeted |
| *H. Influenzae, S. aureus,* Rhinovirus/enterovirus | amoxicillin-clavulanic acid | Adequate | Targeted |
| *H. Influenzae, M. catarrhalis* | piperacillin-tazobactam | Targeted | Targeted |
| *H. Influenzae, P. aeruginosa, S. aureus,*  *K. oxytoca, E. cloacae* | piperacillin-tazobactam | Adequate | Targeted |
| *H. Influenzae, S. aureus* | piperacillin-tazobactam | Adequate | Targeted |
| *H. Influenzae, M. catarrhalis,* Corona virus | amoxicillin-clavulanic acid | Targeted | Targeted |
| *H. Influenzae, S. aureus* | piperacillin-tazobactam, macrolides | Adequate | Adequate |
| *M. catarrhalis, S. aureus, K. oxytoca* | amoxicillin-clavulanic acid | Adequate | # |
| *M. catarrhalis, S. agalactiae* | amoxillin-clavulanic acid | Targeted | # |
| *S. aureus, M. catarrhalis, E. cloacae, Acinetobacter baumanii* | pivmecillinam/pivampicillin/ampicillin | Inappropriate | # |
| *S. aureus, P. aeruginosa* | piperacillin-tazobactam | Adequate | # |
| **Virus with or without enterobacterales (n=19)** | | | |
| *E.coli,* Rhinovirus/enterovirus | ingen beh | # | # |
| Influenzae A | ingen beh | # | # |
| *Proteus* | ingen beh | # | # |
| Parainfluenza virus og Corona virus | ingen beh | # | # |
| *E .coli* | ingen beh | # | # |
| Influenzae A | ingen beh | # | # |
| *E .coli* | ingen beh | # | # |
| Parainfluenza virus | pipetazo | # | # |
| Parainfluenza virus | pipetazo | # | # |
| Corona virus | amoxicillin-clavulanic acid | # | # |
| Rhinovirus/enterovirus | CAVE, macrolides | # | # |
| Acinetobacter baumanii | phenoexythylpenicillin | # | # |
| *K. aerogenes* | piperacillin-tazobactam | # | # |
| Parainfluenza virus | piperacillin-tazobactam | # | # |
| *E. coli* | ingen beh | # | # |
| *S. marcescens* | ingen beh | # | # |
| *K. pneumoniae, K. oxytoca, E.cloacae* | piperacillin-tazobactam | # | # |
| *E. cloacae, K. pneumoniae* | benzylpenicillin | # | # |
| *K. oxytoca*, Respiratory syncytial virus | amoxicillin-clavulanic acid | # | # |
